# Supplementary material for: The Central Nervous System Source Modulates Microglia Function and Morphology In Vitro
Source: Int J Mol Sci. 2023 Apr 22;24(9):7685. doi: 10.3390/ijms24097685 (PMC10177862; doi:10.3390/ijms24097685)
Supplement: Supplementary file 1 [file ijms-24-07685-s001.zip › ijms-2316363-supplementary.pdf]

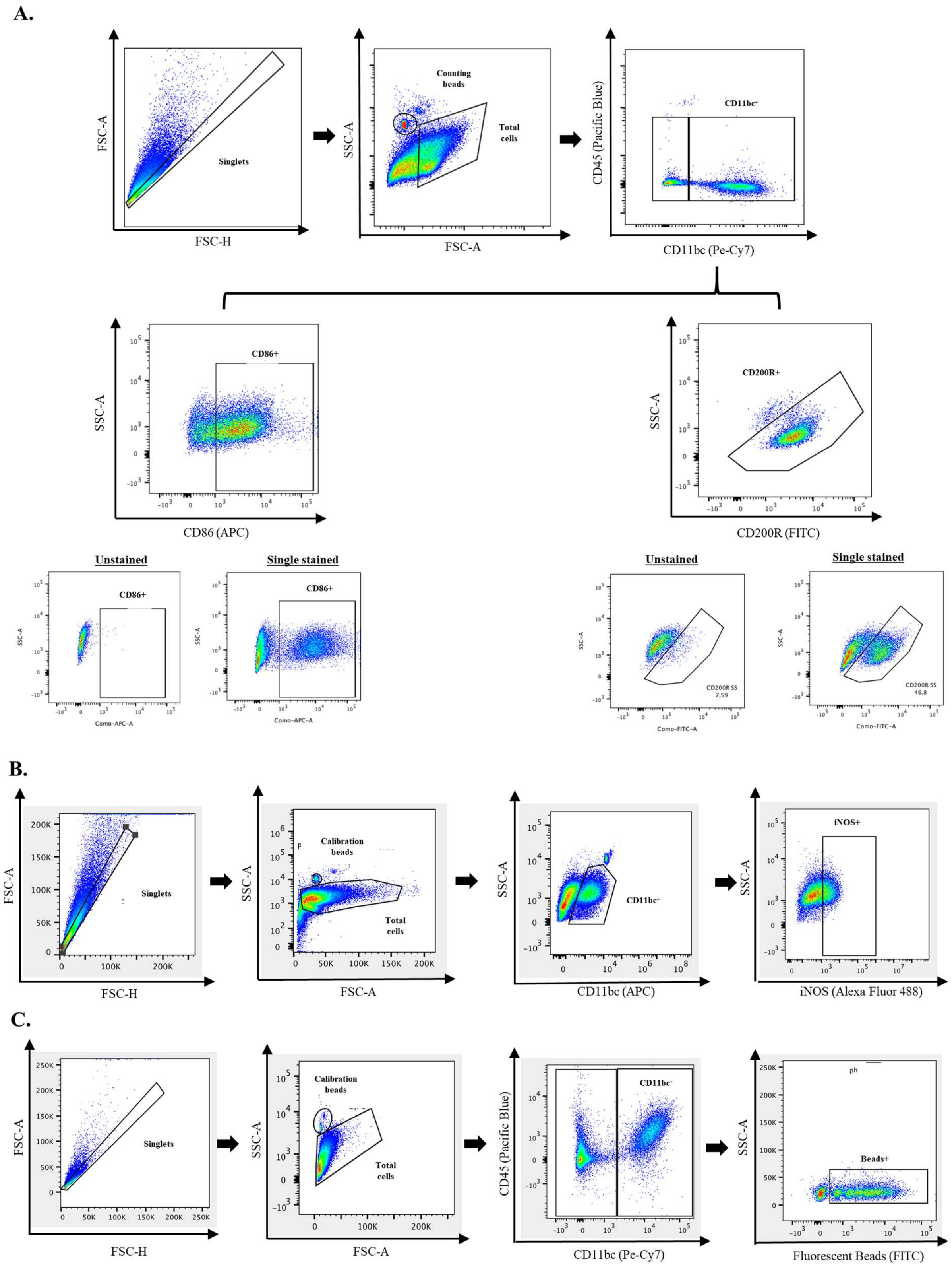

Supplementary Figure S1. Flow cytometry gating strategy A. Gating strategy used for flow cytometry cell number and microglia activation analysis on in vitro glial culture analysis. Doublets were excluded by FSC-A vs FSC-H scatter. Culture total cells were gated by SSC-A vs FSC-A scatter in which it was also selected the counting beads used to calculate cell concentration. Microglial cells were gated by CD45+CD11bc+ cells and on this population CD86^{+} cells and CD200R^{+} cells were defined according to unstained and single stained samples. B. Gating strategy used for flow cytometry microglia iNOS activation analysis. Doublets were excluded by FSC-A vs FSC-H scatter. Culture total cells were gated by SSC-A vs FSC-A scatter. Microglial cells were gated by CD11bc^{+} cells and on this population iNOS^{+} cells were defined according to unstained and single stained samples. C. Gating strategy used for flow cytometry microglia phagocytosis analysis. Doublets were excluded by FSC-A vs FSC-H scatter. Microglial cells were gated by CD45^{+}CD11bc^{+} cells and on this population cells that present beads engulfed were gated as Beads^{+} cells.
